# Availability of Medications for the Treatment of Opioid Use Disorder Among Pregnant and Postpartum Individuals in US Jails

**DOI:** 10.1001/jamanetworkopen.2021.44369

**Published:** 2022-01-20

**Authors:** Carolyn Sufrin, Camille T. Kramer, Mishka Terplan, Kevin Fiscella, Sarah Olson, Kristin Voegtline, Carl Latkin

**Affiliations:** 1 Department of Gynecology and Obstetrics, Johns Hopkins University School of Medicine, Baltimore, Maryland; 2 Department of Health, Behavior, and Society, Johns Hopkins Bloomberg School of Public Health, Baltimore, Maryland; 3 Friends Research Institute, Baltimore, Maryland; 4 Department of Family Medicine, University of Rochester School of Medicine and Dentistry, Rochester, New York; 5 Center for Community Health and Prevention, University of Rochester School of Medicine and Dentistry, Rochester, New York; 6 Biostatistics, Epidemiology, and Data Management Core, Johns Hopkins University School of Medicine, Baltimore, Maryland; 7 Department of Pediatrics, Johns Hopkins University School of Medicine, Baltimore, Maryland

## Abstract

**Importance:**

Thousands of pregnant people with opioid use disorder (OUD) enter US jails annually, yet their access to medications for OUD (MOUD) that meet the standard of care (methadone and/or buprenorphine) is unknown.

**Objective:**

To assess the availability of MOUD for the treatment of pregnant individuals with OUD in US jails.

**Design, Setting, and Participants:**

In this cross-sectional study, electronic and paper surveys were sent to all 2885 identifiable US jails verified in the National Jails Compendium between August 19 and November 7, 2019. Respondents were medical and custody leaders within the jails.

**Main Outcomes and Measures:**

The primary outcome was the availability of MOUD (methadone and/or buprenorphine) for the treatment of pregnant people with OUD in US jails. Availability of MOUD was assessed based on (1) continuation of MOUD for pregnant incarcerated individuals (if the individual was receiving MOUD before incarceration), with or without initiation of MOUD; (2) both initiation and continuation of MOUD for pregnant individuals; (3) only continuation of MOUD for pregnant individuals; and (4) management of opioid withdrawal for pregnant individuals. Secondary outcomes included MOUD availability during the postpartum period and logistical factors associated with the provision of MOUD. Multivariate logistic regression analysis was used to assess factors associated with MOUD availability during pregnancy.

**Results:**

Among 2885 total surveys sent, 1139 (39.5%) were returned; of those, 836 surveys (73.4%; 29.0% of all surveys sent) could be analyzed, with similar proportions from metropolitan (399 jails [47.7%]) and rural (381 jails [45.6%]) settings. Overall, 504 jails (60.3%) reported that MOUD was available for medication continuation, with or without medication initiation, during pregnancy. Of those, 267 jails (53.0%; 31.9% of surveys included in the analysis) both initiated and continued MOUD, and 237 jails (47.0%; 28.3% of surveys included in the analysis) only continued MOUD; 190 of 577 jails (32.9%; 22.7% of surveys included in the analysis) reported opioid withdrawal as the only management for pregnant people with OUD. Among the 504 medication-providing jails, only 120 (23.8%) continued to provide MOUD during the postpartum period. Methadone was more commonly available at jails that only continued MOUD (84 of 123 jails [68.3%]), whereas buprenorphine was more commonly available at jails that both initiated and continued MOUD (73 of 119 jails [61.3%]). In an adjusted model, jails with higher odds of MOUD availability were located in the Northeast (odds ratio [OR], 10.72; 95% CI, 2.43-47.36) or metropolitan areas (OR, 1.92; 95% CI, 1.31-2.83), had private health care contracts (OR, 1.49; 95% CI, 1.03-2.14) and a higher number of women (≥70) reported in the female census (OR, 1.69; 95% CI, 1.02-2.80), and provided pregnancy testing within 2 weeks of arrival at the jail (OR, 2.66; 95% CI, 1.69-4.17).

**Conclusions and Relevance:**

In this cross-sectional study, a substantial proportion of US jails did not provide access to MOUD to pregnant people with OUD. Although most jails reported continuing to provide MOUD to individuals who were receiving medication before incarceration, few jails initiated MOUD, and most medication-providing jails discontinued MOUD during the postpartum period. These results suggest that many pregnant and postpartum people with OUD in US jails do not receive medication that is the standard of care and are required to endure opioid withdrawal, signaling an opportunity for intervention to improve care for pregnant people who are incarcerated.

## Key Points

**Question:**

What is the availability of medications for opioid use disorder (MOUD) for pregnant people in US jails?

**Findings:**

In this cross-sectional study of 836 survey respondents from jails across the US, respondents at 60% of jails reported continuing to provide MOUD to pregnant individuals who were receiving medication before incarceration, but only 32% of jails initiated MOUD during pregnancy. Most medication-providing jails discontinued MOUD during the postpartum period.

**Meaning:**

This study found that US jails did not consistently provide pregnant people with access to medications that meet the standard of care for treatment of opioid use disorder, which suggests that there is an opportunity for intervention to improve care for pregnant people who are incarcerated.

## Teaser

This cross-sectional study uses verified data from the National Jail Compendium to assess the availability of medication for the treatment of opioid use disorder among pregnant individuals incarcerated in US jails.

## Introduction

Incarcerated individuals and institutions of incarceration in the US have experienced and played a role in the broad-reaching and fatal consequences of the opioid epidemic. The burden of opioid use disorder (OUD) is high among people in jail, with one-quarter to one-third of the 2 million people with OUD cycling in and out of jails each year.^1,2^ Compared with the general population, incarcerated people with OUD are more than 100 times more likely to die of opioid overdoses when they return to their communities, in part because of reduced opioid tolerance associated with untreated OUD while in custody.^3,4,5^ Despite increasing recognition of the important role of carceral facilities in providing medications for OUD (MOUD) to incarcerated individuals, little research has addressed the needs of pregnant people with OUD in jails.

It has been estimated that among the nearly 55 000 admissions of pregnant people to jails each year, approximately 7700 individuals (14%) have OUD.^6,7^ Rates of OUD during pregnancy quadrupled in the US between 1999 and 2016, with fatal consequences; by 2016, 10% of deaths among pregnant and postpartum individuals were associated with opioid use.^8,9,10,11^ This problem impacts not only pregnant individuals but their children, families, and communities. Pregnant people with substance use disorders are often criminalized rather than given access to treatment.^12^ They encounter intersecting forms of discrimination and stigma from being incarcerated, pregnant, and using drugs. Structural racism undergirds the marginalization of pregnant people who use drugs, with policies that disproportionately penalize pregnant Black individuals and mandate separation from their children.^12,13,14,15^

The well-established standard of care for OUD during pregnancy is MOUD consisting of methadone or buprenorphine.^16^ Maternal and fetal benefits include increases in adherence to addiction treatment, prenatal care, and in-hospital delivery and decreases in the risk of overdose death, HIV, hepatitis, preterm birth, and infants with low birth weight.^17,18,19,20^ Medically supervised opioid withdrawal is not recommended during pregnancy and has been associated with return to opioid use, producing fetal and maternal risks.^18^ One study^19^ found that incarceration during pregnancy or the postpartum period was associated with 4 times greater odds of postpartum overdose, whereas receiving MOUD during pregnancy was protective. For pregnant people in jail, the potential benefit of providing MOUD is substantial, combining the established benefits of medication both during pregnancy and while in custody. Provision of MOUD to people in custody has been associated with reductions in fatal and nonfatal overdoses, decreases in involuntary opioid withdrawal, improvements in engagement with addiction treatment, reductions in reincarceration rates, and increases in employment rates.^21,22,23,24,25,26,27,28^

Amid national calls to expand access to MOUD in carceral settings, little is known about the availability of these medications in jails.^29^ One reason for this gap is the large number of jails in the US for which no publicly available database exists. Jails differ from prisons on many levels, including the fact that most people in jails, which are locally administered, have pretrial status and will return to their communities after a short stay. This fluidity between jails and the community highlights the importance of MOUD availability in jails as a point along the cascade of care.^30^ We conducted the first study, to our knowledge, to use verified data from the National Jails Compendium.^31^ The study involved a national cross-sectional survey of all identifiable jails in the US to assess practices regarding MOUD and opioid withdrawal among pregnant people at those facilities.

## Methods

### Participants

We administered a cross-sectional survey between August 19 and November 7, 2019, to medical and custody leaders at all 2885 US jails verified by the National Jails Compendium,^31^ the first known database containing information on the contact persons and mailing addresses of all known US jails. We specified that the survey should be completed by someone knowledgeable about the jail’s MOUD services. The Johns Hopkins School of Medicine Institutional Review Board approved the study, with respondents' completion of the survey serving as their informed consent to participate in the study. This study followed the Strengthening the Reporting of Observational Studies in Epidemiology (STROBE) reporting guideline for cross-sectional studies.^32^

### Survey Instrument and Outcomes

Our primary outcome was the availability of MOUD at jails (termed MOUD-available jails). We first identified whether MOUD (methadone and/or buprenorphine) was available to pregnant individuals for continuation of medication (if the individual was receiving MOUD before incarceration), with or without initiation of medication, among the 836 jails included in the analysis. We then subdivided MOUD-available jails into those that both initiated and continued MOUD and those that only continued but did not initiate MOUD. Jails reporting that they provided only naltrexone during pregnancy were not classified as MOUD-available jails because the use of naltrexone is generally not recommended during pregnancy.^16^ The survey was adapted from a previous study on prison access to MOUD during pregnancy^7^ and consisted of 49 items pertaining to methadone and/or buprenorphine for the treatment of pregnant people with OUD, opioid withdrawal practices during pregnancy, MOUD dosing arrangements, and access to MOUD in the postpartum period. Other characteristics included health care delivery arrangements, pregnancy testing practices, the position and/or title of the person completing the survey, and the number of women and pregnant people at the facilities based on a female census item included in the survey (with June 30, 2019, as the date of census).

### Recruitment and Survey Distribution

The National Sheriff’s Association endorsed our study, and we promoted this support in recruitment materials. We first sent electronic surveys to those with an email address (2039 jails [70.7%]) using REDCap (Johns Hopkins University), a secure web-based platform (Figure).^33^ We then mailed surveys via US Postal Service to those without an email address and those who did not respond to the online survey after 2 reminders. Recruitment strategies have been published elsewhere.^31^ Jails were considered nonrespondents if their surveys were not received by March 31, 2020.

**Figure.**
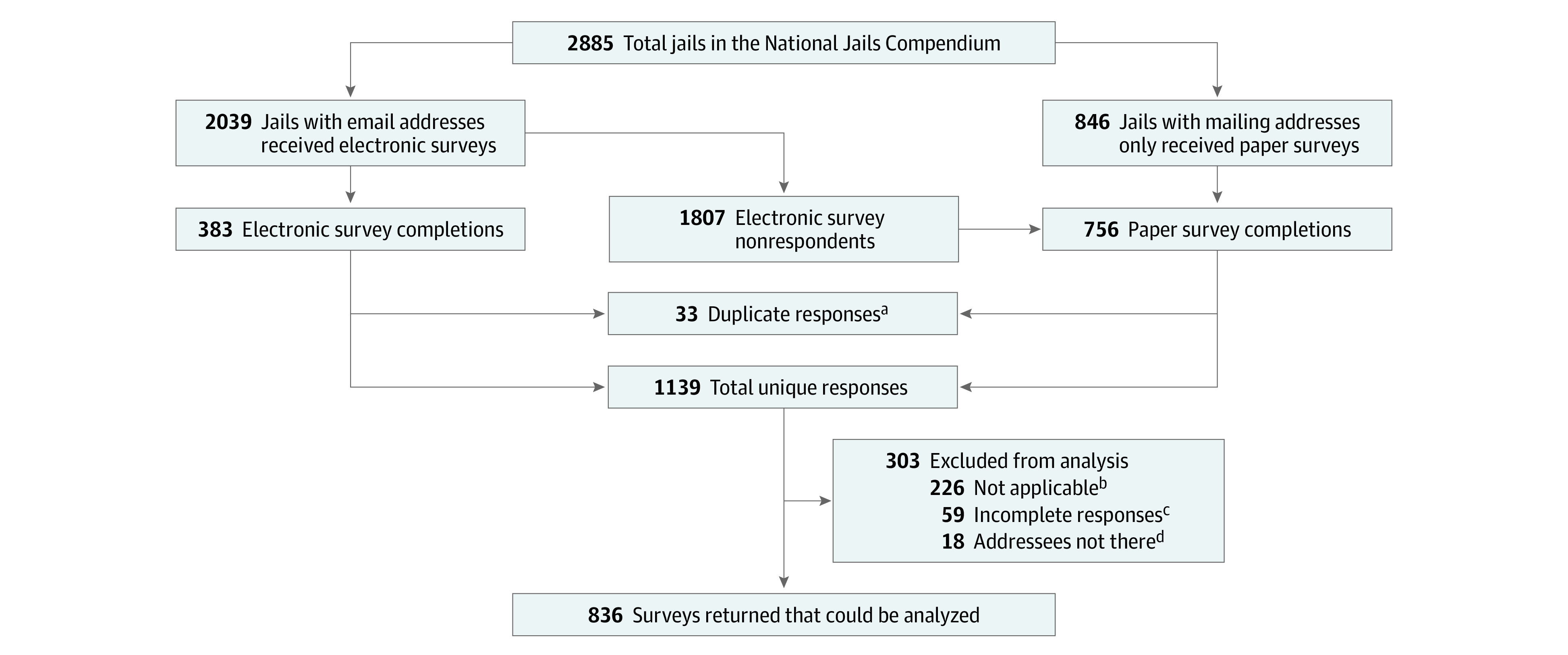
Recruitment, Response, and Surveys That Could Be Analyzed Among All Known US Jails ^a^True duplicate responses were defined as more than 1 response (complete or partially complete) from the same address. ^b^Respondents indicated the survey was not appropriate for their jail setting. Sample reasons included short-term holding facility, no jail at the location, no women (and/or pregnant individuals) housed at the jail, and all detainees were sent to another jail. ^c^Surveys that included no information about the primary outcome. ^d^Individuals who responded to the survey by communicating that the person to whom the survey was addressed was not at that location.

### Data Management

Electronic surveys were completed by respondents using REDCap, and paper surveys sent by respondents were manually entered into REDCap by study team members. Surveys were excluded from the analyses if they were duplicates (ie, more than 1 response [complete or partially complete] from the same address), were from a jail setting that was not applicable for this study (ie, jails that did not house pregnant people), or were incomplete (ie, included no information associated with the study’s primary outcome) (Figure).

We encountered discrepancies in some participants’ responses to survey items pertaining to our main outcome, MOUD availability; these discrepancies were mainly found in paper surveys. For instance, some respondents reported that they did not offer methadone during pregnancy but then provided details on how they administered methadone. Therefore, we developed an algorithm to adjudicate discrepancies. If a respondent provided credible responses regarding how methadone or buprenorphine was administered, the facility was considered an MOUD-available jail. Discrepancies that could not, after review by 3 raters (C.S., C.T.K., and C.L.), clarify MOUD availability were categorized as MOUD-uncertain jails and were not included in the primary analysis of MOUD-available jails. Surveys from respondents at MOUD-uncertain jails generally consisted of those reporting that MOUD was available during pregnancy without subsequent valid responses regarding how MOUD was provided. We then conducted 2 sensitivity analyses, one in which MOUD-uncertain jails were reclassified as MOUD-available jails and another that classified MOUD-uncertain jails as withdrawal only. We did not apply the adjudication process to opioid withdrawal responses and reported opioid withdrawal as those who selected withdrawal on the survey.

### Statistical Analysis

We analyzed outcomes and important variables using descriptive statistics and applying Pearson χ^2^ or Wilcoxon rank sum tests. Observations with missing outcomes were excluded from denominators for proportion calculations. We used zip codes to categorize jail locations as metropolitan or rural.^34^ We used respondents’ reported positions and/or titles to categorize them as having vs not having decision-making capacity and as having custody vs medical roles. Univariate logistic regression models were used to identify jail characteristics and practices of MOUD-available jails. Significant factors were included in a multivariate model. Multivariate models were repeated for the provision of both methadone and buprenorphine. Before conducting multivariate modeling, we confirmed that survey type (paper vs online) was not significantly associated with main outcomes. All analyses were performed using SAS software, version 9.4.(SAS Institute Inc), and the significance threshold was 2-sided *P* < .05.

## Results

 Of 2885 surveys sent, we received 1139 unique responses (383 electronic and 756 paper), representing a response rate of 39.5% (Figure). A total of 836 surveys (29.0% of all surveys sent and 73.4% of survey responses) could be analyzed. Overall, 1048 of 1746 nonrespondents (60.0%) were from rural counties, with a greater proportion from the South and Midwest regions; 586 nonrespondents (33.6%) had a physical address only. The 836 respondents with surveys that could be analyzed were from jails representing a broad geographic range, with similar distribution of metropolitan (399 jails [47.7%]) and rural (381 jails [45.6%]) settings (Table 1). Most respondents who provided information about their position or title had a custody role (654 of 820 respondents [79.8%]) or a decision-making role (509 of 820 respondents [62.1%]). Few jails (259 of 836 [31.0%]) conducted routine pregnancy testing at intake or within 2 weeks of arrival. The most common health care delivery arrangement was through contracts with private correctional health care companies (384 of 836 jails [45.9%]). The 1-day median count of pregnant people across all included jails on June 30, 2019, was 1506 (range, 0-65 people per facility), or 3.0% of all women (N = 50 248) in study jails.

**Table 1.** Characteristics of Respondents and Jails

Characteristic	No. /total No. (%)
Total respondents, No.	836
Region	
West	169/836 (20.2)
Midwest	282/836 (33.7)
South	314/836 (37.6)
Northeast	71/836 (8.5)
Geographic classification^a^	
Metropolitan	399/836 (47.7)
Rural	381/836 (45.6)
Role of survey respondent	
Custody	654/820 (79.8)
Health care	166/820 (20.2)
Decision-making	509/820 (62.1)
Non–decision-making	311/820 (37.9)
Women reported at female census on June 30, 2019, median (IQR)^b^	29 (10-69.5)
Pregnant women reported on June 30, 2019, median (IQR)	1 (0-2)
Pregnancy testing policies	
No testing performed under any circumstances	46/836 (5.5)
All individuals tested at intake	193/836 (23.1)
All individuals tested within 2 weeks of arrival but not at intake	66/836 (7.9)
Individuals tested only at practitioner or individual request	524/829 (63.2)
Routine prenatal care service delivery	
On-site care from a health care professional (physician, certified nurse-midwife, nurse practitioner, or physician assistant)	345/816 (42.3)
Off-site care only	422/816 (51.7)
Other care only	49/816 (6.0)
Health care service delivery^c^	
Contract with private correctional health care company^d^	384/836 (45.9)
Public agency	108/836 (12.9)
University health care center contract	24/836 (2.9)
Individual health care professional	127/836 (15.2)
Direct through jail	147/836 (17.6)
Community health care professional, clinic, hospital, or community nonprofit organization	227/836 (27.2)
Other^e^	75/836 (9.0)

^a^ Geographic classification was based on zip code and National Center for Health Statistics urban and rural county categories criteria.^34^ Metropolitan includes large metropolitan urban, large metropolitan suburban, and medium to small metropolitan areas. A total of 56 respondents (6.7%) had a missing zip code.

^b^ Online responses had a greater median female census than paper responses (32.5 vs 25.0; *P* = .04).

^c^ Categories were not mutually exclusive; therefore, percentages total greater than 100%.

^d^ Privately contracted health care was reported less frequently among online responses than paper responses (151 vs. 233; *P *= .01).

^e^ Health care services delivery arrangements with other organizations or health care professionals were more common among paper responses than they were among online responses (64 vs 11; *P* < .001).

Overall, among 836 jails included in the analysis, 504 (60.3%) met our criteria for having a high certainty of continuing MOUD during pregnancy (Table 2). A total of 267 jails (31.9% of all jails included in the analysis and 53.0% of MOUD-available jails) both initiated and continued MOUD, and 237 jails (28.3% of all jails included in the analysis and 47.0% of MOUD-available jails) continued but did not initiate MOUD during pregnancy. Among all 836 jails, 190 (22.7%) reported managing OUD only through opioid withdrawal. An additional 142 jails (17.0%) reported that MOUD was available during pregnancy, but details were missing or contradictory (MOUD-uncertain jails).

**Table 2.** Availability of Medication for the Treatment of Opioid Use Disorder Among Pregnant Individuals in US Jails

MOUD availability	Jails, No./total No. (%)
**During pregnancy**	
MOUD available^a^	504/836 (60.3)
Continuation only	237/504 (47.0)
Initiation and continuation	267/504 (53.0)
Methadone available	385/504 (76.4)
Continuation only	247/385 (64.2)
Initiation and continuation	137/385 (35.6)
Buprenorphine available	381/504 (75.6)
Continuation only	171/381 (44.9)
Initiation and continuation	210/381 (55.1)
Methadone only available	123/504 (24.4)
Continuation only	84/123 (68.3)
Initiation and continuation	39/123 (31.7)
Buprenorphine only available	119/504 (23.6)
Continuation only	46/119 (38.7)
Initiation and continuation	73/119 (61.3)
Both methadone and buprenorphine available	262/504 (52.0)
Continuation only	107/262 (40.8)
Initiation and continuation	152/262 (58.0)
Withdrawal only (no MOUD available)^b^	190/577 (32.9)
Withdrawal and MOUD available^c^	387/577 (67.1)
**During postpartum period**	
MOUD available for continuation^d^	120/504 (23.8)
No MOUD continuation	274/504 (54.4)
Discontinuation with tapering	165/274 (60.2)
Abrupt discontinuation	61/274 (22.3)
Conditional discontinuation^e^	47/274 (17.2)
Did not report	110/504 (21.8)

Abbreviation: MOUD, medication for opioid use disorder.

^a^ Data do not include jails for which the provision of MOUD was uncertain.

^b^ There was a higher likelihood of doing withdrawal only among paper responses than among online responses (126 vs. 64; *P* = .01).

^c^ There was a higher likelihood of doing withdrawal and MOUD among paper responses than among online responses (267 vs. 120; *P* = .03).

^d^ Among jails providing MOUD during pregnancy.

^e^ Some respondents stated that they did not routinely continue providing MOUD after pregnancy but would consider allowing it depending on the remaining time in an individual’s jail sentence or whether an individual was expressing breast milk.

Jails that only provided methadone were more likely to continue medication only (84 of 123 jails [68.3%]) than to initiate and continue medication (39 of 123 jails [31.7%]). In contrast, jails that only provided buprenorphine were more likely to initiate and continue medication (73 of 119 jails [61.3%]) than to continue medication only (46 of 119 jails [38.7%]) (Table 2). A total of 262 jails (52.0% of MOUD-available jails) provided access to both methadone and buprenorphine, and 76 jails (15.1% of MOUD-available jails) routinely switched from 1 medication to the other when providing MOUD to pregnant individuals. Although naltrexone is not recommended during pregnancy,^16^ 141 jails (28.0% of MOUD-available jails) reported providing naltrexone to pregnant individuals.

In the first sensitivity analysis, when MOUD-uncertain jails were classified as MOUD-available jails, the overall proportion of facilities providing MOUD increased from 504 of 836 (60.3%) to 646 of 836 (77.3%; *P* < .001). In the second sensitivity analysis, when MOUD-uncertain jails were classified as jails managing OUD only through opioid withdrawal, the overall proportion of jails managing OUD only through withdrawal increased from 131 of 577 (22.7%) to 229 of 577 (39.7%; *P* < .001). Although only 14 of 141 respondents (9.9%) at MOUD-uncertain jails had a medical role, 124 of 494 respondents (25.1%) at MOUD-available jails had a medical role (*P* < .001). A similar proportion of respondents at MOUD-available and MOUD-uncertain jails had decision-making roles (315 of 494 respondents [63.8%] and 89 of 141 respondents [63.1%], respectively; *P* = .24). Respondents at several jails wrote in their own responses, reporting that they managed OUD during pregnancy by releasing people from jail (8 respondents) or transferring them to another jail or prison at which they could have access to medications (12 respondents).

Few of the 504 MOUD-available jails continued MOUD during the postpartum period (120 jails [23.8%]) (Table 2). Among 274 MOUD-available jails (54.4%) that discontinued MOUD after pregnancy, discontinuation practices ranged from abrupt cessation (61 jails [22.3%]) to tapering (165 jails [60.2%]) of medication. Some respondents reported that continuation of MOUD during the postpartum period was conditional (47 jails [17.2%]) based on whether the person had a short interval until expected jail release (or transfer to another facility) or whether the person was breastfeeding or expressing breast milk.

The most common dispensing arrangement among 386 jails providing methadone was picking up or sending medication to the jail for administration by jail staff (205 jails [53.1%]) followed by transporting the pregnant person off site to receive medication (199 jails [51.6%]) (Table 3). Of 386 jails providing buprenorphine, most (262 jails [67.9%]) dispensed medication on site, with fewer (96 jails [24.9%]) transporting the pregnant person off site to receive medication. Among 577 jails reporting information on opioid withdrawal during pregnancy, most withdrawals occurred on site (472 jails [81.8%]). Although most jails (437 [75.7%]) provided medications for symptom relief during withdrawal, 28 jails (4.9%) did not. A total of 114 jails (19.8%) used opioids other than methadone or buprenorphine for withdrawal management.

**Table 3.** Medication for Opioid Use Disorder, Logistical Details of Medication Provision, and Opioid Withdrawal Arrangements Among US Jails Providing Medication for the Treatment of Pregnant Individuals With Opioid Use Disorder

Arrangement	Jails, No. (%)			
	Type of MOUD		Opioid withdrawal	
	Methadone	Buprenorphine	Reported that opioid withdrawal was used for pregnant people	Did not provide information about opioid withdrawal practices for pregnant people
**MOUD provision**				
Total jails, No.	137	210	NA	NA
Location of treatment initiation^a^^,^^b^				
Jail	47 (34.3)	145 (69.0)	NA	NA
Hospital	31 (22.6)	51 (24.3)		
Other community site	72 (52.6)	45 (21.4)		
Other	74 (54.0)	49 (23.3)		
**Logistical details of MOUD provision** ^**a**^ **^,^ ^c^**				
Total jails, No.	386	386	NA	NA
On-site dispensing (certified OTP or buprenorphine treatment professional with waiver)	15 (3.9)	262 (67.9)	NA	NA
Transportation to community site for dosing	199 (51.6)	96 (24.9)		
Medication brought to jail by OTP staff	63 (16.3)	53 (13.7)		
Medication picked up or sent to jail for jail staff to administer	205 (53.1)	17 (4.4)		
Other	50 (13.0)	51 (13.2)		
**Opioid withdrawal**				
Total jails, No.	NA	NA	577	259^d^
Location of withdrawal^a^				
Jail	NA	NA	472 (81.8)	NA
Hospital			136 (23.6)	NA
Transfer to another jail or release from custody			20 (3.5)	NA
Other^e^			49 (8.5)	NA
Withdrawal practices during pregnancy				
Abrupt withdrawal only	NA	NA	28 (4.9)	NA
Withdrawal with medication support^a^			437 (75.7)	NA
Withdrawal with nonopioid medications for symptoms			303 (52.5)	NA
Withdrawal with opioids not used as MOUD^f^			114 (19.8)	NA
Withdrawal with methadone or buprenorphine for symptoms only			131 (22.7)	NA
Not reported			112 (19.4)	NA

Abbreviations: MOUD, medication for opioid use disorder; NA, not applicable; OTP, opioid treatment professional.

^a^ Categories are not mutually exclusive; therefore, percentages do not total 100%.

^b^ Initiation site is only reported for jails that initiated treatment with methadone and/or buprenorphine.

^c^ Logistical details apply to initiation and/or continuation of MOUD, whichever the jail had available. The jails were not asked about logistical details for continuation or initiation of MOUD separately.

^d^ Includes 142 jails for which the provision of MOUD was uncertain because they did not provide information about opioid withdrawal.

^e^ One statistically significant difference between paper and online responses was found, with more paper responses selecting the *other* category.

^f^ Includes acetaminophen with codeine and acetaminophen with hydrocodone (eg, Norco and Vicodin).

We used logistic regression analysis to identify jail characteristics associated with MOUD availability during pregnancy (Table 4). In the unadjusted multivariate model, facilities with higher odds of providing MOUD during pregnancy were located in the Northeast (odds ratio [OR], 5.66; 95% CI, 2.31-13.85; *P* < .001) or metropolitan settings (OR, 2.89; 95% CI, 2.15-3.89; *P* < .001), had a higher number of women (≥70) reported in the female census (OR, 3.42; 95% CI, 2.33-5.03; *P* < .001), provided routine pregnancy testing within 2 weeks of arrival (OR, 4.27; 95% CI, 3.00-6.09; *P* < .001), and used private health care contractors for care delivery (OR, 2.07; 95% CI, 1.56-2.76; *P* < .001). Jails with lower odds of providing MOUD during pregnancy were located in the South (OR, 0.66; 95% CI, 0.44-0.97; *P* < .001) and comprised survey respondents who were employed in a custody role (OR, 0.44; 95% CI, 0.30-0.65; *P* < .001).

**Table 4.** Factors Associated With Availability of Medication for the Treatment of Opioid Use Disorder During Pregnancy in US Jails

Factor	OR (95% CI)	*P* value
**Unadjusted model of factors associated with provision of MOUD**		
Census region		
Midwest vs West	0.62 (0.42-0.92)	<.001
Northeast vs West	5.66 (2.31-13.85)	
South vs West	0.66 (0.44-0.97)	
Geographic classification		
Metropolitan vs rural	2.89 (2.15-3.89)	<.001
Health care service delivery		
Private vs not private	2.07 (1.56-2.76)	<.001
Role of survey respondent		
Custody vs health care	0.44 (0.30-0.65)	<.001
Decision-making vs non–decision-making	1.20 (0.90-1.60)	.22
Female census ≥70 vs <70 women^a^	3.42 (2.33-5.03)	<.001
Pregnancy test within 2 wk of arrival (yes vs no)	4.27 (3.00-6.09)	<.001
On-site vs off-site prenatal care	1.51 (1.12-2.04)	.007
**Adjusted model of factors associated with provision of MOUD^b^**		
Census region		
Midwest vs West	0.62 (0.38-1.02)	<.001
Northeast vs West	10.72 (2.43-47.36)	
South vs West	0.54 (0.33-0.89)	
Geographic classification		
Metropolitan vs rural	1.92 (1.31-2.83)	<.001
Health care service delivery		
Private vs not private	1.49 (1.03-2.14)	.03
Role of survey respondent		
Custody vs health care	0.54 (0.33-0.87)	.011
Female census ≥70 vs <70 women^a^	1.69 (1.02-2.80)	.04
Pregnancy testing within 2 wk of arrival (yes vs no)	2.66 (1.69-4.17)	<.001
On-site vs off-site prenatal care	1.03 (0.72-1.48)	.87
**Adjusted model of factors associated with provision of both methadone and buprenorphine**		
Census region		
Midwest vs West	0.86 (0.48-1.51)	.09
Northeast vs West	1.57 (0.76-3.22)	
South vs West	0.68 (0.40-1.16)	
Geographic classification		
Metropolitan vs rural	2.01 (1.23-3.20)	.004
Role of survey respondent		
Custody vs medical	0.90 (0.56-1.43)	.64
Health care service delivery		
Private vs not private	1.31 (0.87-1.98)	.19
Female census ≥70 vs <70 women^a^	0.93 (0.56-1.54)	.79
Pregnancy testing within 2 wk of arrival (yes vs no)	0.76 (0.49-1.12)	.24
On-site vs off-site prenatal care	1.03 (0.68-1.57)	.89

Abbreviations: MOUD; medication for opioid use disorder; OR, odds ratio.

^a^ The cutoff for small vs large jails was set at 70 women based on the IQR of the female census counts reported by respondents.

^b^ The sensitivity analysis using the model in which MOUD-uncertain jails were categorized as MOUD-available jails revealed that only the South had lower odds of providing MOUD (OR, 0.45; 95% CI, 0.26-0.82) vs the West, and jails that performed routine pregnancy testing within 2 weeks of arrival had higher odds of providing MOUD (OR, 2.54; 95% CI, 1.58-4.26) vs those that did not.

When we adjusted the multivariate model to categorize MOUD-uncertain jails as MOUD-available jails, facilities with routine pregnancy testing within 2 weeks of arrival (OR, 2.66; 95% CI, 1.69-4.17; *P* < .001), facilities located in the Northeast (OR, 10.72; 95% CI, 2.43-47.36; *P* < .001) or metropolitan areas (OR, 1.92; 95% CI, 1.31-2.83; *P* < .001), and facilities that had private health care contracts (OR, 1.49; 95% CI, 1.03-2.14; *P* = .03) and a greater number of women recorded in the female census (OR, 1.69; 95% CI, 1.02-2.80; *P* = .04) continued to have higher odds of providing MOUD, and facilities located in the South (OR, 0.54; 95% CI, 033-0.89; *P* < .001) continued to have lower odds of providing MOUD. In the second adjusted multivariate model including jails with both methadone and buprenorphine available, only those located in a metropolitan setting had higher odds of providing MOUD (OR, 2.01; 95% CI, 1.23-3.20; *P* = .004) (Table 4).

## Discussion

This cross-sectional study is, to our knowledge, the largest study to date of US jails using verified data from the National Jails Compendium.^31^ The study found that a substantial proportion of jails did not provide access to MOUD, which is the standard of care, to pregnant people with OUD. Overall, 39.7% of jails did not allow continuation of MOUD during pregnancy, and only 31.9% permitted initiation of MOUD during pregnancy. This finding suggests that thousands of pregnant people entering jails are likely required to endure opioid withdrawal each year.^7^ Withdrawal is not only a difficult experience, especially when in custody, but it is also accompanied by short- and long-term risks for both pregnant people and their fetuses, including increased risk of overdose combined with higher risk of overdose at jail release and greater risk of suicide.^4,18,19,21,35,36^ In addition, allowing a pregnant person to experience opioid withdrawal during pregnancy increases the need for staff to respond to medical issues resulting from withdrawal. However, 60.3% of jails provided continuation of MOUD during pregnancy, suggesting that implementation of MOUD is feasible across a range of jail sizes and locations.

Providing methadone to individuals in custody requires complex logistical arrangements because a jail has to either have a licensed opioid treatment program or devise arrangements with a community organization to transport individuals or medications, often on a daily basis. Treatment with buprenorphine, in contrast, requires only a practitioner with an X waiver to prescribe.^37^ In our study, methadone and buprenorphine were similarly available overall, but jails that only provided continuation of MOUD were more likely to do so using methadone only; jails that initiated and continued MOUD were more likely to provide buprenorphine than methadone. This difference may reflect the different logistical issues associated with medication administration. If a jail employs a buprenorphine prescriber who has a waiver, it can easily initiate and continue MOUD on site. If a jail only provides access to methadone, it is likely to do so only for people who were receiving MOUD before incarceration.

The standard of care for pregnant people with OUD is access to both methadone and buprenorphine and the ability to choose (with practitioner guidance) the medication that works best. However, only 152 jails (18.2% of all jails included in the analysis and 30.2% of MOUD-available jails) provided access to initiation and continuation of MOUD with both medications. It is concerning that 54.4% of MOUD-available jails discontinued MOUD during the postpartum period. This practice implies that the benefit of MOUD is solely for the fetus, overlooks the importance of the mother’s long-term well-being for herself and her infant, and does not reflect standard practices for chronic disease management of OUD. Furthermore, postpartum cessation of MOUD is known to increase the risk of fatal and nonfatal overdose.^8,9,19^

The provision of MOUD during pregnancy was less common in smaller rural jails and jails not located in the Northeast, which is consistent with patterns in the national availability of MOUD.^38,39,40^ Jails without routine pregnancy testing were also less likely to provide MOUD, reflecting an absence of standardized pregnancy care. Jails with routine pregnancy testing may also be more attentive to the distinct needs of this population. Jails that had privately contracted health care were more likely to provide MOUD, which may reflect the contractors’ awareness of national guidelines and their desire to reduce medicolegal risks. Compared with a national survey of 245 jails conducted 20 years ago,^41^ in which 15% of jails continued providing methadone during pregnancy, there has been modest improvement.

Previous survey studies of prisons^29,42^ found that most prisons providing MOUD do so exclusively during pregnancy, although details on initiation or continuation of MOUD were not reported; these studies^29,42^ may have cultivated a misperception that pregnant people in custody have greater access to MOUD than nonpregnant individuals.^43^ A more recent (2020) study^7^ of 22 state prison systems and 6 jails found that, although most systems continued providing MOUD to individuals who were receiving medication before incarceration, only 4 prison systems would initiate MOUD during pregnancy; likewise, of 6 jails in that study, only 2 initiated MOUD. The fact that few prisons initiate MOUD during pregnancy highlights the importance across the incarceration spectrum of jails providing MOUD. Because most prisons only continue but do not initiate MOUD,^7^ it is important that pregnant individuals receive these medications while in jail and before arrival at prison.

The high proportion of jails that did not provide MOUD during pregnancy in our study was similar to the proportion found in a survey study of 53 jails,^44^ in which at least 46% of jails reported opioid withdrawal, not access to MOUD, as their means of managing OUD among pregnant individuals. In addition, among 179 pregnant people with OUD in the North Carolina prison system, which includes pretrial detention, almost one-half of individuals endured opioid withdrawal.^45^

The lack of required health care standards and oversight in carceral facilities, despite a constitutional requirement to provide health care, has produced substantial variability in available health care services,^46,47^ which is consistent with our findings of variable access to MOUD during pregnancy. Furthermore, with more than 3000 jails in the US, most of which are under local jurisdiction, some jail leaders may not have the resources to obtain information or enact standards of care. Lawsuits have established that nonprovision of MOUD violates the Americans with Disabilities Act along with other legal precedents and that carceral facilities must make MOUD available^48^; nonetheless, practice lags behind legal and ethical standards.^21^

Jails are short-stay facilities in which people are temporarily detained after arrest; thus, they are largely responsible for the immediate management of treatment for people with OUD within the incarceration system. Jails have direct continuity with the community because people frequently come and go. Whether pregnant people receive MOUD or experience opioid withdrawal while in jail has direct implications for their well-being and survival in both the facility and the community.

Thus, providing MOUD to pregnant people in jails is an important strategy for optimizing maternal and infant health. Moreover, given racial and ethnic disparities in incarceration rates, providing MOUD during pregnancy to those in custody is also important for addressing racial and ethnic inequities in maternal health. Providing links to care via established partnerships with community organizations and health care professionals is equally important. Continuation of MOUD requires communication between the jail and the community organization or health care professional at jail entry, and continuing MOUD after leaving jail requires early and active planning to ensure continuity of medications. Increased resources, practice changes, standardization and oversight, and links to care are necessary for jails to provide access to MOUD during pregnancy. Such actions are needed to improve pregnancy outcomes, mitigate the intergenerational harms of the opioid epidemic, and address maternal health inequities.

### Limitations

This study has several limitations. These include the representativeness of the sample. Although the 39.5% response rate may appear low, it is substantially higher than the 14% response rate reported from a phone survey of 384 jails.^44^ Potential reasons for nonresponses from jail personnel could include apprehension or disinterest in participating in research studies and thinking such research would not be useful to them.^31^ Notably, nonresponses reveal the challenges of conducting jail-based research that could prevent progress in this area. Most nonrespondents were from rural counties and the South and Midwest regions of the US; based on respondents’ answers, provision of MOUD was lower among jails in the South and Midwest, suggesting that response bias likely overestimates the proportion of jails offering MOUD during pregnancy.

We did not assess the ways in which jails consider individual preferences for medication, and we did not ask about screening practices for OUD. The fact that we could not be certain about MOUD availability for 17.0% of responses highlights the difficulties of ascertaining consistent information about health care services among jails. Findings from the sensitivity analysis suggested that MOUD-uncertain jails were different from jails in which MOUD ascertainment was certain, which supported our strict adjudication process for discrepancies. Future research could investigate which individuals actually received counseling and which individuals wanted and received MOUD during pregnancy. Researchers could also interview administrators, especially those at jails with few resources, about the challenges of providing care for this population and interview pregnant people with OUD who are incarcerated.

## Conclusions

This cross-sectional study found that a substantial proportion of US jails did not provide access to MOUD to pregnant and postpartum individuals. Although respondents at most jails reported that they continued to provide MOUD to pregnant individuals who were receiving medication before incarceration, few jails initiated MOUD, and most medication-providing jails discontinued MOUD during the postpartum period. These results suggest that many pregnant and postpartum individuals with OUD in US jails do not receive MOUD that meets the standard of care and are often required to endure opioid withdrawal, signaling an opportunity for intervention to improve care for pregnant individuals who are incarcerated.
